# A language-independent hearing screening self-test at school-entry

**DOI:** 10.1038/s41598-024-53026-y

**Published:** 2024-01-31

**Authors:** Elien Van den Borre, Gaziz Tufatulin, Lea Zupan, Nina Božanić Urbančič, Limor Lavie, Inga Holube, Vinay Swarnalatha Nagaraj, Emre Gurses, Sam Denys, Astrid van Wieringen, Jan Wouters

**Affiliations:** 1 Department of Neurosciences, Research Group ExpORL, KU Leuven, Herestraat 49 Bus 721, 3000 Leuven, Belgium; 2 Center of Pediatric Audiology, St Petersburg, Russia; 3 North-Western State Medical University Named After I.I.Mechnikov, St Petersburg, Russia; 4 Scientific Research Institute of Ear, Nose, Throat and Speech, St Petersburg, Russia; 5 Department of Ear, Nose, and Throat, General Hospital Celje, Celje, Slovenia; 6 Department of Otorhinolaryngology and Cervicofacial Surgery, University Medical Centre Ljubljana, Ljubljana, Slovenia; 7 Faculty of Medicine, Department of Otorhinolaryngology, University of Ljubljana, Ljubljana, Slovenia; 8 Department of Communication Sciences and Disorders, University of Haifa, Haifa, Israel; 9 Institute of Hearing Technology and Audiology, Jade University of Applied Sciences, Oldenburg, Germany; 10 Audiology Group, Department of Neuromedicine and Movement Sciences, Norwegian University of Science and Technology, Trondheim, Norway; 11 Department of Audiology, Faculty of Health Science, Hacettepe University, Ankara, Turkey; 12 Department of Otorhinolaryngology-Head and Neck Surgery, Multidisciplinary University Center for Speech-Language Pathology and Audiology, University Hospitals of Leuven, Leuven, Belgium

**Keywords:** Health care, Public health

## Abstract

The usage of a tablet-based language-independent self-test involving the recognition of ecological sounds in background noise, the Sound Ear Check, was investigated. The results of 692 children, aged between 5 and 9 years and 4 months, recruited in seven different countries, were used to analyze the validity and the cultural independence of test. Three different test procedures, namely a monaural adaptive procedure, a procedure presenting the sounds dichotically in diotic noise, and a procedure presenting all the sounds with a fixed signal-to-noise ratio and a stopping rule were studied. Results showed high sensitivity and specificity of all three procedures to detect conductive, sensorineural and mixed hearing loss > 30 dB HL. Additionally, the data collected from different countries were consistent, and there were no clinically relevant differences observed between countries. Therefore, the Sound Ear Check can offer an international hearing screening test for young children at school entry, solving the current lack of hearing screening services on a global scale.

## Introduction

About two to three in 1000 children are born with hearing loss^1^, and about the same number acquire hearing loss during early childhood^2^. Untreated hearing loss increases the risk for speech, language, and learning difficulties and low social-communicative abilities^3–8^. Early intervention has proven highly effective in reducing these adverse effects^9–13^ and therefore, cost-effectiveness ratios of childhood hearing screening are estimated to be extremely high^14–16^. Many middle- and high-income countries have implemented newborn hearing screening (NHS) programs to ensure early detection and rehabilitation of congenital hearing loss, and a third of these NHS programs capture data from at least 85% of newborns^17^. However, late-onset, progressive, and acquired hearing losses present themselves at a later stage. Consequently, despite the installation of NHS, a significant number of children are still at risk for the numerous negative side effects of untreated childhood hearing loss^3–8^. Therefore, the World Health Organization (WHO) and the European Federation of Audiology Societies (EFAS) strongly recommend screening all children for hearing loss and ear diseases at school entry as a bare minimum^15,18^. However, in most countries, systematic school-age hearing screening (SHS) does not exist beyond NHS^2,19^, and if at all, data on the few installed SHS projects are poorly documented, the protocols and practices are inconsistent, and loss of follow-up of the referred children is an ubiquitous concern^14^. As a result, novel and inventive approaches are required to meet the recommendations of the WHO and EFAS and provide qualitative, standardized protocols for childhood hearing screening.

For a couple of years, the EFAS workgroup for childhood hearing screening has been working on a new and innovative approach to providing one standardized test method that can solve the lack of childhood hearing screening in many countries. This novel methodology, utilizing Signal-in-Noise testing as its foundation, forms a solution to the challenges systematic childhood hearing screening currently faces. The test of interest, named the ‘Sound Ear Check’ or ‘SEC’, is a closed-set, tablet-based self-test based on the perception of sounds, such as the barking of a dog or the honking of a car, chosen to have similar temporal and spectral features as natural speech, covering the frequency range that is most important for speech understanding^20,21^. A pilot study with the SEC in adults showed a high correlation with pure tone audiometry, the golden standard in audiology, and the Digit Triplet Test (DTT)^22^, a widely used speech understanding test used for hearing screening and diagnostics^23,24^. The SEC is a self-test operated on a standard digital device such as a tablet with standard headphones, reducing the material cost largely and making a trained test leader no longer required. Additionally, due to the language independence of the SEC's sound material, one version can be used across countries, which brings additional costs and scientific resources needed for translation studies back to zero. Therefore, the SEC methodology may be an easily implementable, language-independent, low-cost alternative with similar results to, for example, the widely used DTT^22^.

In the current international study, the final version of the SEC was developed and validated. This test version was optimized for the application in young children, implementing elements such as animated drawings and automatic test progression to increase the attention and motivation of the children tested. This study investigated and compared the sensitivity and specificity of the SEC for normal-hearing (NH) children and children with various degrees of conductive (CHL), sensorineural (SNHL) and mixed hearing loss (MHL) with a standard monaural adaptive procedure, such as tested in the pilot study^22^, a procedure with a fixed signal-to-noise ratio (SNR) and a stopping rule that has the potential to strongly decrease the duration of the test^25^, and a binaural antiphasic procedure, i.e. a procedure where the noise is presented without phase difference between ears and the stimuli with a phase difference of 180° between ears, that can half the duration and increase the sensitivity and specificity of the test for various types of hearing loss^26,27^. Moreover, this research investigated whether, when used in a wide variety of countries, cultural differences affected the sound-reception threshold (SoRT)-values, their standard deviation (SD) as a measure of the stability of the test, and the familiarity of the sounds. This study used data from seven countries that cover a broad range of cultures, including Belgium, Slovenia, Russia, Germany, Norway, Israel, and Turkey, to deliver a language-independent hearing screening self-test for school-entry that can solve the lack of hearing screening in many countries.

## Methods

### Participants

**Figure 1:**
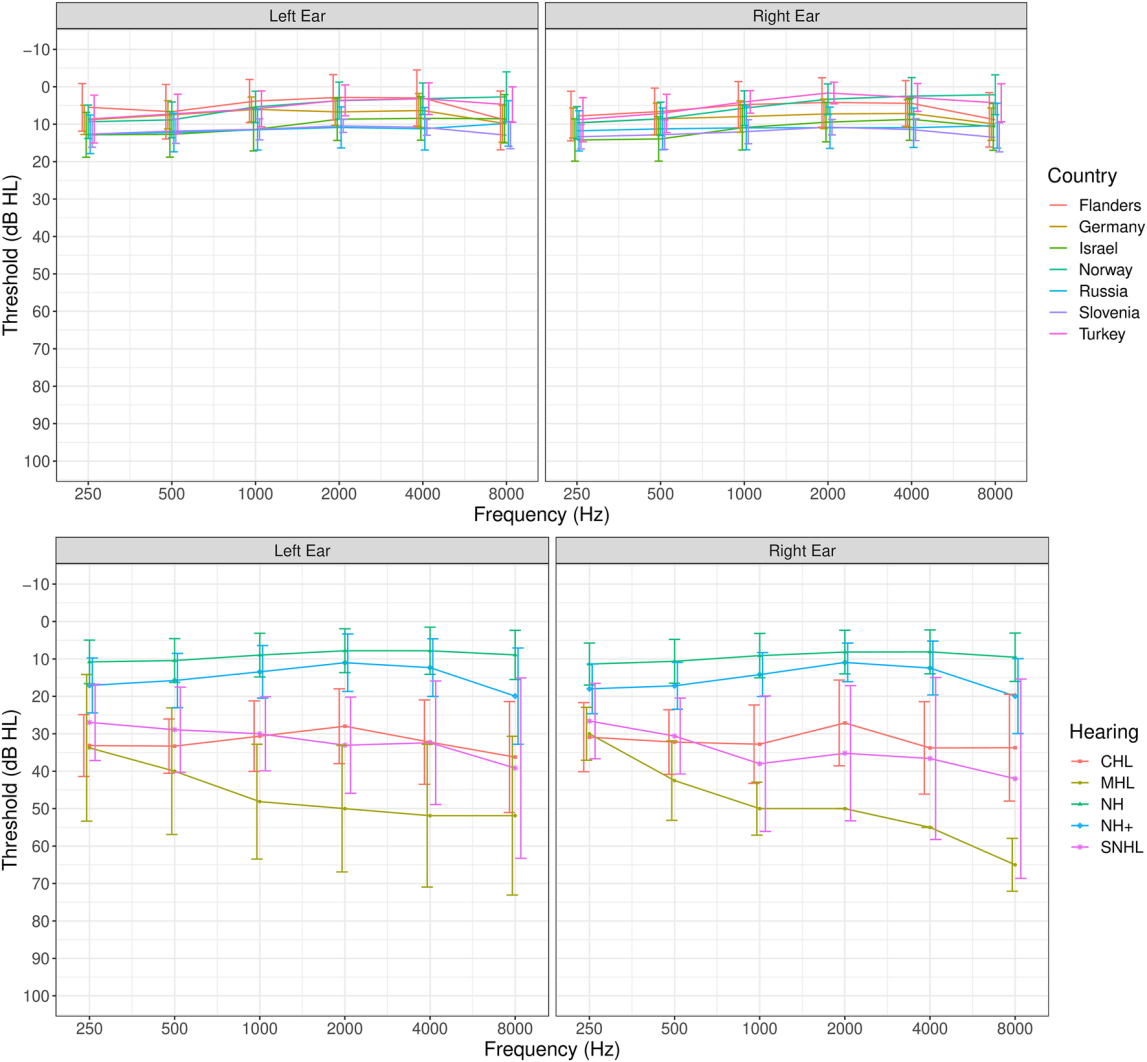
Average audiogram per ear and SD of NH children per country (**a**) and of all children (NH, CHL, SNHL, MHL) per hearing group (**b**).

NH and hard of hearing (HoH) children aged between 5 and 9 years and 4 months (mean: 7 years 1 month ± 10 months) were recruited. The parent(s) and/or legal guardian(s) of the participating children received a detailed explanation and signed an informed consent form before participation. A simplified informed consent explaining the important aspects of the research was signed by the participating children. For the SEC with the monaural adaptive procedure (SEC_REF_) and the SEC with the monaural fixed procedure (SEC_FIX_), ears were considered separately, meaning the same child could be included in different hearing loss groups. This means that a possible asymmetry in hearing is not considered for the analyses of the results of the SEC_REF_ and SEC_FIX_. However, as the sounds used in the SEC have spectral and temporal features similar to speech, the model of Plomp (1978)^28^ can be used to estimate the possibility of crossover hearing affecting the results. The noise in the SEC_REF_ and SEC_FIX_ is fixed at 65 dB SPL. When speech is presented through air conduction, the interaural attenuation would be 40 dB SPL, meaning that the noise would be heard at 25 dB SPL in the other ear. According to the model of Plomp (1978)^28^, the SRTs of normal hearing people start worsening when the noise is presented at 30 dB SPL or lower. Therefore, even when the better ear overhears the stimuli and the noise without any distortion during the transmission, the results would still deviate from the norm value and the hearing loss would still be detected. If the better ear is influenced by the worse ear, a screening test should be able to detect this problem so that the patient can be referred for diagnostics. The diagnostic phase should then identify what causes the hearing problem and how to best rehabilitate it. For the antiphasic procedure (SEC_APH_), both ears were considered together, and children were divided into groups based on the hearing in both ears (Table 1). Ears were considered NH when the air conduction (AC) pure-tone average (PTA)_0.5–4 kHz_ was < 20 dB HL and all thresholds from 250 to 8000 Hz were < 20 dB HL. Ears with a PTA_0.5–4 kHz_ < 20 dB HL, but one or more thresholds ranging from 250 to 8000 Hz ≥ 20 dB HL were considered as NH+, as depending on the configuration of the hearing loss, the effects on the SoRT-values obtained with the SEC versions can be very heterogenous, which asks for a more detailed analysis. Ears were considered to have CHL when an AC PTA_0.5–4 kHz_ ≥ 20 dB HL, a bone conduction (BC) PTA_0.5–4 kHz_ < 20 dB HL, and an Air Bone Gap (ABG) ≥ 10 dB were present. Ears were considered to have SNHL when an AC PTA_0.5–4 kHz_ ≥ 20 dB HL, a BC PTA_0.5–4 kHz_ ≥ 20 dB HL, and an ABG < 10 dB were present. Ears were considered to have MHL when an AC PTA_0.5–4 kHz_ ≥ 20 dB HL, a BC PTA_0.5–4 kHz_ ≥ 20 dB HL, and an ABG ≥ 10 dB were present. When a child had a hearing loss > 80 dB HL in their worse ear, the results on the SEC_APH_ were removed from the statistical analysis, as well as the results of SEC_REF_ and the SEC_FIX_ in that ear specifically, as it would be impossible to hear the noise and stimuli when such hearing loss was present. The number of children included per country, the number of ears/children included per SEC-test, and the average PTA_0.5–4 kHz_ per hearing group and per SEC version are given in Table 1. In total, data from 692 children were collected. Two hundred fifty-eight were female, 281 were male, and of 153 children, no data was available on their sex. In 111 of the cases, a different mother tongue was spoken by the child than the official language of the country where they were living. In 101 of these cases, the child was NH. The average audiogram and SD for the different hearing groups and the average audiogram of NH ears per country are presented in Fig. 1.

**Table 1:** Number of ears tested with different test versions per hearing group and their average PTA_0.5–4 kHz_ between brackets.

Number of children tested per country										
Total	Flanders	Slovenia	Russia	Germany	Turkey	Norway	Israel			
692	91, NH: 67	143, NH: 63	184, NH: 144	67, NH: 59	41, NH: 25	44, NH: 43	122, NH: 101			

Reference procedure (n ears)										
Total	NH	NH+	CHL	SNHL	MHL					
1210	896 (9 ± 5 dB HL)	164 (13 ± 5 dB HL)	92 (31 ± 9 dB HL)	48 (33 ± 13 dB HL)	10 (48 ± 14 dB HL)					

Fixed procedure (n ears)										
494	363 (9 ± 5 dB HL)	61 (14 ± 4 dB HL)	42 (34 ± 10 dB HL)	23 (33 ± 13 dB HL)	5 (42 ± 6 dB HL)					

Antiphasic procedure (children)										
Total	NH-NH	NH-NH+	NH+ -NH+	NH-CHL	CHL-CHL	NH-SNHL	CHL-SNHL	SNHL-SNHL	MHL-SNHL	NH+-MHL
313	225 (10 ± 5 dB HL)	5 (13 ± 5 dB HL)	35 (15 ± 5 dB HL)	17 (28 ± 8 dB HL)	12 (34 ± 6 dB HL)	3 (27 ± 9 dB HL)	2 (33 ± 5 dB HL)	10 (39 ± 15 dB HL)	3 (42 ± 6 dB HL)	1 (60 dB HL)

### Materials

All audiometers used were calibrated according to ISO standards. The following audiometers were used: Madsen Orbiter 922 (*Flanders*), Madsen Midimate 622 (*Flanders*), Auritec AT-1000 (*Germany*), Auritec AT-900 (*Germany*), GSI Audiostar PRO (*Turkey*), Maico MA 52 (*Israel*), Interacoustics AD 229e (*Israel*), Entomed SA-201 (*Norway*), Interacoustics Clinical AC40 (*Slovenia*), AD226 (*Russia*), and a GSI-61 audiometer (*Russia*). Transducers similar or equal to RadioEar DD65 embedded in peltor caps were used when testing outside of an audio booth. Transducers similar or equal to Telephonics TDH39 were used for testing inside of an audio booth.

The SEC was performed on a 7-inch Samsung Galaxy Tab A tablet connected to RadioEar DD65 transducers embedded in Peltor caps. RadioEar DD65 transducers yield a damping of ~ 30–40 dB SPL on the frequencies important for speech understanding. The test setup was calibrated with SWN at 80 dB Sound Pressure Level (SPL) with a Brüel & Kjaer Sound level meter 2260 and a Brüel & Kjaer 4153 artificial ear using the flat plate. The study was designed and conducted according to the Declaration of Helsinki. This study was approved by the Ethics Committee Research UZ/KU Leuven. All methods were performed in accordance with the relevant guidelines and regulations.

### Sound ear check versions

**Figure 2:**
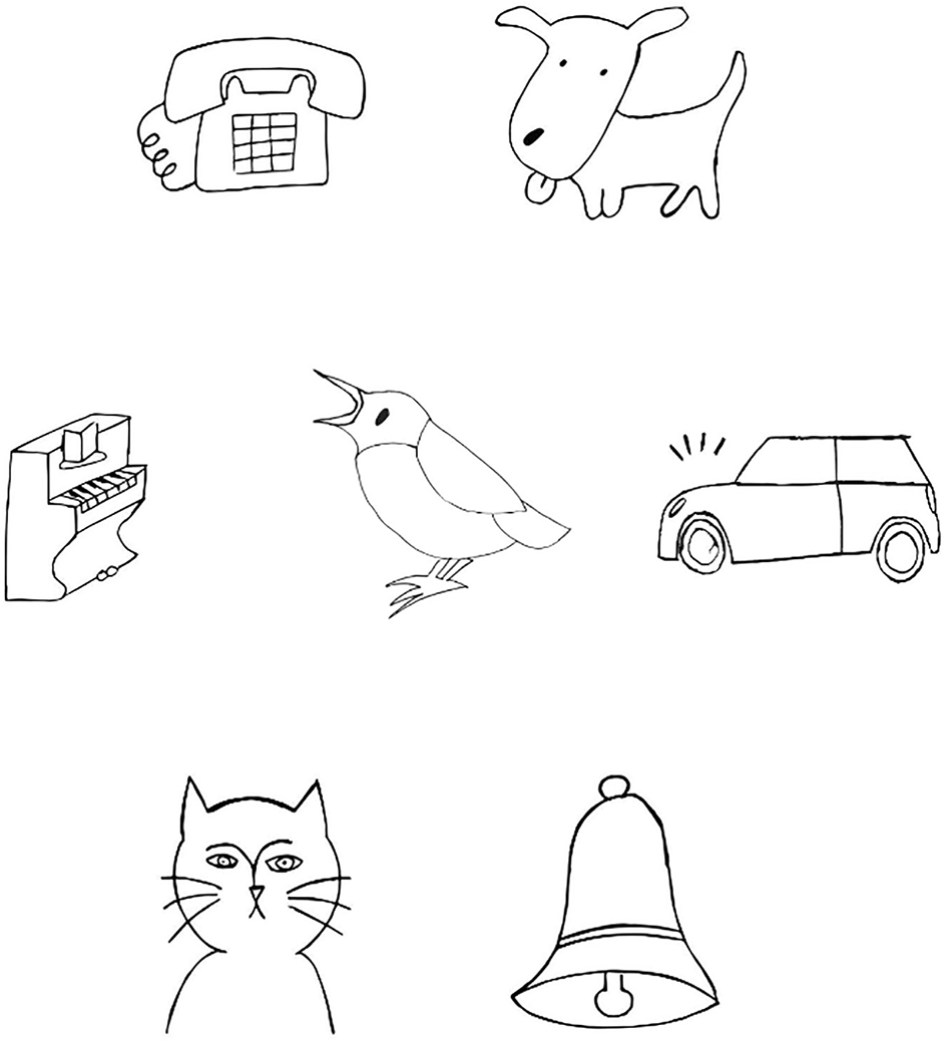
Interface of the SEC. Drawings correspond to the sounds used. Drawings were chosen based on recognizability.

Three different test procedures were used. The interface of the SEC was the same for all procedures (Fig. 2). All SEC versions used seven sounds presented in sound-weighted noise (SWN), i.e., noise with a spectrum identical to the average spectrum of the test materials^22^. Seven animated drawings, chosen based on recognizability, were shown on the tablet screen (Fig. 2). The sounds, including barking of a dog, honking of a car, a ringing phone, a playing piano, ringing bells, mewing of a cat, and chirping of a bird, were chosen based on recognizability and spectral and temporal features, which were kept as close as possible to the features of natural speech^22^. All procedures consisted of three subsequent phases. The first phase, the acclimatization phase, was the same for all procedures. In this phase, the sounds were presented diotically at a fixed SNR of 0 dB SNR and the noise fixed at 65 dB SPL. Each sound was presented in random order until identified correctly, after which the image disappeared from the screen as a sign that the sound-image mapping was correct. The fixed SNR of 0 dB SNR was chosen so that children can get used to more challenging, but most likely not impossible, SNRs before the start of the actual training and test phases. During these phases the images did not disappear after a correct answer, so the participant did not receive direct feedback. A fixed SNR of 0 dB SNR can be too challenging for children with more severe hearing loss. However, as the SEC is meant to be a screening test, it does not target children with severe hearing loss but children with undetected hearing loss, and this is most likely not severe. Additionally, in the case of an undetected severe hearing loss, the inability to perform the test indicates hearing loss with a referral for diagnostics as a result.

For **SEC**_**REF**_**,** the second phase was a diotic training phase consisting of 21 trials with every sound played three times (3 × 7 sounds) in random order. The training phase used an adaptive procedure in which the stimuli were adapted in steps of 2 dB, and the noise was fixed at 65 dB SPL. A fixed noise level of 65 dB SPL can be challenging for participants with severe hearing loss. However, as the SEC is designed to be a screening test, it targets people with undetected, and therefore most likely mild to moderate hearing loss. For these people, 65 dB SPL is still suprathreshold. In the case of an undetected severe hearing loss, the inability to perform the test will again result in a referral for diagnostics. For the first seven trials, a 1-up-1-down procedure was used to converge fast to the SoRT. A 1-up-2-down procedure was used for the subsequent trials, targeting a recognition probability of 71% correct^29^. The third phase was the actual test phase, which used 21 trials per ear and the same procedure as the training phase but with monaural stimulus presentation, first testing the left ear, then the right ear. The contralateral ear was not masked during the test phase to avoid binaural unmasking, which could cause additional, unwanted variability in the SRT-values. The SoRT was calculated by averaging the SNR of the last 15 trials, including a non-presented (imaginary) 22nd trial, of which the SNR was calculated based on the identification response of the final presented item. A SEC_REF_ result was considered reliable when the SD of the 15 trials used for the calculation of the SoRT-value was < 2.5 dB SNR.

The **SEC**_**APH**_ presented antiphasic stimuli, i.e. with a phase difference of 180° between ears, in in-phase noise. This presentation mode relied on binaural unmasking or Binaural Masking Level Differences (BMLD), which is an improvement of the SoRT or speech reception threshold in noise due to a phase difference of the stimuli between both ears. BMLDs are reported to be poorer for listeners with all types of hearing loss than for NH controls, which makes the difference in performance between NH and HoH participants larger^30^. For the SEC_APH_, the acclimatization phase was followed by two dichotic training phases with 21 trials and one dichotic test phase with 21 trials, as previous research suggested that a longer training phase was needed when testing children dichotically^31^. The training and the test phases used the same adaptive procedure as described for the SEC_REF_ but with a starting SNR of − 10 dB SNR and noise fixed at 70 dB SPL because a BMLD of ± 10 dB SNR was expected^26,27,31^. The SEC_APH_ result and its reliability were calculated as described for the SEC_REF_.

The **SEC**_**FIX**_ used two short binaural training phases with a variable number of trials (max. seven trials per phase) and two monaural test phases with a variable number of trials (max. 21 trials per ear). The noise was fixed at 65 dB SPL. The stopping rule calculated a preliminary proportion correct for each trial. The proportion-correct was compared to a predefined proportion of 90%. The test was stopped when either the pass or fail proportion was higher than the predefined value or when the maximal number of trials was reached. The result was no longer an SoRT but a pass/fail classification. The SNR used during the first training phase was − 4 dB SNR, and the SNR used during the second training phase was − 7.8 dB SNR, the optimal cut-off value to differentiate between NH children and children with CHL > 30 dB HL and SNHL > 20 dB HL with the SEC_REF_^31^. In the training phases, the stopping rule could only result in an early fail result, and NH children who performed well would always complete the entire training needed to avoid motivation loss for children that do not hear any of the sounds at this SNR but still give NH children enough training to proceed to the test phase. After the training phase, a monaural test phase was performed for each ear with the stopping rule described above, but this time the test could result in both an early fail and an early pass. During the test phase, the SNR was again − 7.8 dB SNR, the optimal cut-off to differentiate between NH children and children with CHL > 30 dB HL and SNHL > 20 dB HL as estimated in previous research^31^. The contralateral ear was not masked during the test phase to avoid binaural unmasking, which could cause additional, unwanted variability in the SRT-values.

### Protocol

All children were tested in a quiet room (at home or in school) or an audio booth (at the hospital). Noise floors in quiet rooms were not measured as the used headphones (RadioEar DD65) give enough damping (~ 30–40 dB SPL) to attenuate the average noise levels in quiet rooms (~ 30 dB SPL). The total protocol (audiometry + two SEC versions + 5-min break) took 40 to 50 min. Most children started with the SEC tests unless the circumstances did not allow it. Every child did the SEC_REF_, combined with the SEC_FIX_ or the SEC_APH_. All SEC tests were done as a self-test, meaning the child performed the test autonomously. The order in which the SEC tests were done was randomized, resulting in four possible sequences, named ‘RA’, ‘AR’, ‘RF’, and ‘FR’, which consist respectively of the SEC_REF_ and SEC_APH_, the SEC_APH_ and SEC_REF_, the SEC_REF_ and SEC_FIX_, and the SEC_FIX_ and SEC_REF_. When the time was available, the protocol could be extended with the third test version that was not done yet. If, due to circumstances such as a time restriction, a reliable second test was not possible, only one SEC version was done/included. The hearing thresholds (250–8000 Hz) were measured using the Hughson-Westlake method. If an AC threshold was worse than 20 dB HL at one or more frequencies, the BC thresholds for those frequencies were measured. Masking was used according to standardized guidelines^32^.

### Statistical analyses

Statistical analyses were performed using R and R Studio^33^. For the general analyses and the analyses on the cultural dependence of the SEC, only the data of NH children was used. Values of children/ears with a PTA_0.5–4 kHz_ < 20dB HL but one or more thresholds worse than 20 dB HL (NH+) were not included in the analyses described below unless specified differently. The analyses done with the data of children with NH+ were described separately in the last part of the paragraph of the statistical analyses section.

#### General analyses

A possible age effect on the SoRT-values obtained with the SEC_APH_ was estimated with a simple linear model (LM) with 'age' as the independent variable and 'SoRT-value' as the dependent variable. For the SEC_REF_ this effect was estimated with a Linear Mixed Effect Model (LMEM) as two values were obtained per person (left and right ear). The model included 'age' as the independent variable, 'SoRT-value' as the dependent variable and 'ID' as the random effect. For the SEC_FIX_, this effect was estimated with a mixed logistic regression model with 'age' as the independent variable, ‘score’ on the SEC_FIX_ as the dependent variable and ID added as a random effect. The same type of model with 'sex' as the independent variable was used to estimate the effect of ‘sex’ on the SoRT-values obtained with the SEC_REF_, the SEC_APH_ and SEC_FIX_. If an age effect was present, 'age' was added as an additional independent variable. Differences in SoRT-values on the SEC_APH_ obtained in different sequences were estimated with LMs, with ‘SoRT-value’ as the dependent variable and 'sequence’ as the independent variable. For the SoRT-values obtained with the SEC_REF_, an LMEM was used with the same variables, but 'ID' was added as a random effect, and the sequences were grouped based on whether the SEC_REF_ was done first (RA & RF) or last in the sequence (FR or AR). For the SEC_FIX_, a log-linear regression was used with ‘sequence' as the independent variable, 'ID' as the random effect and ‘score’ on the SEC_FIX_ as the dependent variable. The analyses on the effect of ‘sequence’ did not include the results of the extra tests done as an additional third test (see Protocol).

#### Cultural- and language-dependence of the SEC

Differences in SoRT-values on the SEC_REF_ and their SDs obtained in different countries were estimated with an LMEM, with ‘SoRT-value’ or ‘SD’ as the dependent variable and ‘country’ as the independent variable, 'ID' as a random effect. If an age effect was present, 'age' was added as the additional independent variable. The SD of the SoRT-values was used as a measure for the stability of the test. The same type of model with 'mother tongue' instead of 'country' as an independent variable was used to estimate the effect of mother tongue on the SoRT-values obtained with the SEC_REF_. Stimulus–response confusion matrices were made for every country with the values of the SEC_REF_ to estimate the familiarity of the sounds used in different countries. The overall recognition coefficient was compared between countries using a chi-squared test. Differences between countries in specific diagonal scores of the confusion matrices per sound were analyzed with a loglinear regression model with 'country' and 'sound' as independent variables and 'identification' as the dependent variable.

#### Sensitivity and specificity of the optimized SEC-procedures.

The correlation between the PTA_0.5–4 kHz_ and the SoRT-values of the SEC_REF_ was determined with an LMEM with ‘SoRT-values’ as the dependent variable and ‘PTA_0.5–4 kHz_’ as independent variables. ‘ID’ was added as a random effect, and 'age' was added as an additional independent variable if an effect of age was present. The correlation between the PTA_0.5–4 kHz_ and the SoRT-values of the SEC_APH_ was calculated with an LM with ‘SoRT-values’ as the dependent variable and ‘PTA_0.5–4 kHz_’ as the independent variable. A possible interaction effect between the type of hearing loss and the PTA_0.5–4 kHz_ on the SoRT-values of the SEC_REF_ was estimated with a LMEM including only the SoRT-values of the HoH children with the interaction effect between ‘hearing loss type’ and ‘PTA_0.5–4 kHz_’ as independent variable and ‘SoRT-values’ as the dependent variable. ‘ID’ was added as a random effect, and 'age' was added as an additional independent variable in case an age effect was present. For the SoRT-values of the SEC_APH_, the same variables were used in an MLM without ‘ID’ as a random effect. In this analysis, the results of the child with MHL in one ear and NH+ in the other were not considered, as the model does not allow single-value groups.

Sensitivity and specificity to detect hearing losses with a PTA_0.5–4 kHz_ > 20dBHL, > 30dB HL, and > 40 dB HL with the SEC_REF_ and SEC_APH_ were estimated with Receiver Operating Characteristic (ROC) analyses, constructed for CHL and SNHL separately, and for CHL, SNHL and MHL together. ROC curves and the associated sensitivity and specificity were classified according to the principles of Metz^34^. For the SEC_APH_, the hearing type of the worse ear was used to define the overall hearing. No independent analyses were done for MHL, as only a limited number of children with MHL were tested. The assumptions of ROC analyses, namely that the measurement of interest is continuous with an independent diagnosis, that the state variable is independent of the measurement of interest and that the cases are a random sample, were all met. Sensitivity and specificity to detect CHL > 30 dB HL or SNHL > 20 dB HL with the SEC_FIX_ were investigated as simple percentages of correct classification as both values are factors. Symmetric and asymmetric hearing losses were considered together to determine optimal pass-fail criteria for both hearing loss types.

#### Minimal hearing loss

The results of children with NH+ were analyzed separately to get an overview of how the SEC captures these types of minimal hearing loss. The results of the SEC_REF_ and SEC_FIX_ of children with NH+ could have been obtained by children with NH+ in one ear and a more severe hearing loss in the other ear. However, the better ear of these participants was analyzed (the ear with NH+). Therefore, crossover hearing is unlikely to have influenced the results of the ears with NH+. The percentage of pass and fail results based on the ROC analyses described above was calculated to analyze the results of the children in the NH+ group. Afterward, these children were divided into groups based on their results on the SEC_REF_, SEC_APH_, and SEC_FIX_. Possible differences in the audiograms of children with fail results on each test were compared using t-tests. For the SEC_REF_ and the SEC_FIX_, ears were considered separately. For the SEC_APH_, the worst and the best threshold at a certain frequency were considered in separate groups.

## Results

**Figure 3:**
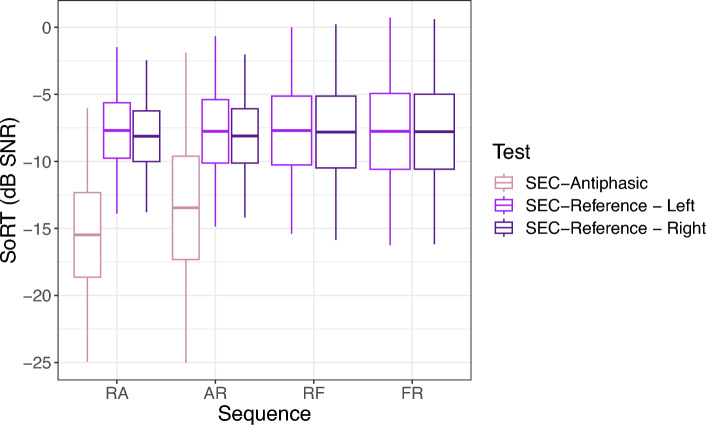
Boxplots showing the mean SoRT-values and 3*SD of NH children for the SEC_REF_ and SEC_APH_ per sequence. For the SEC_REF_, SoRTs are shown for all sequences separately. Sequences ‘RA’, ‘AR’, ‘RF’, and ‘FR’, consist respectively of the SEC_REF_ and SEC_APH_, the SEC_APH_ and SEC_REF_, the SEC_REF_ and SEC_FIX_, and the SEC_FIX_ and SEC_REF._

The average SoRT-values per sequence for NH children for the SEC_REF_ and the SEC_APH_ are given in Fig. 3. Figure 3 does not include age as a variable, which results in slight deviations in the differences between the tests done in different sequences from what is estimated with the statistical analyses.

The average SoRT-values for NH children for the SEC_REF_ and the SEC_APH_ were − 7.9 ± 2.4 dB SNR and − 14.4 ± 3.7 dB SNR, respectively. A significant age effect was present on the results of NH on the SEC_REF_ (t (478.0) = − 5.9, *p* < 0.001) but not on the SEC_FIX_ (z = 1.0, *p* = 0.318) or on the SEC_APH_ (t (3.7, 223) = − 1.3, *p* = 0.208). The regression coefficients indicated that the SoRT-values of the SEC_REF_ decreased with ± 0.7 dB SNR per year. No effect of sex was present on the results of the SEC_REF_ (t (411.7) = − 0.3, *p* = 0.783), on the SEC_APH_ (t (3.8, 192) = − 1.9, *p* = 0.066) or on the SEC_FIX_ (z = − 0.5, *p* = 0.617). For the SEC_REF_ and the SEC_FIX_, results estimated in different sequences did not differ significantly (SEC_REF_: t (488,1) = 0.07, *p* = 0.942, SEC_FIX_: z = 1.9, *p* = 0.056), but for the SEC_APH_, a significant difference of 2.0 dB SNR was present between the SEC_APH_ done first and the SEC_APH_ done second (t (3.5, 221) = − 4.3, *p* < 0.001).

### Cultural- and language-dependence of the SEC

**Figure 4:**
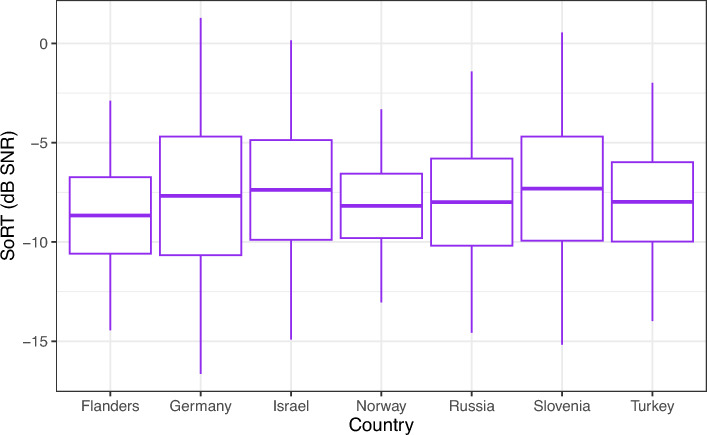
SoRT-values of NH children for the SEC_REF_ per country.

**Figure 5:**
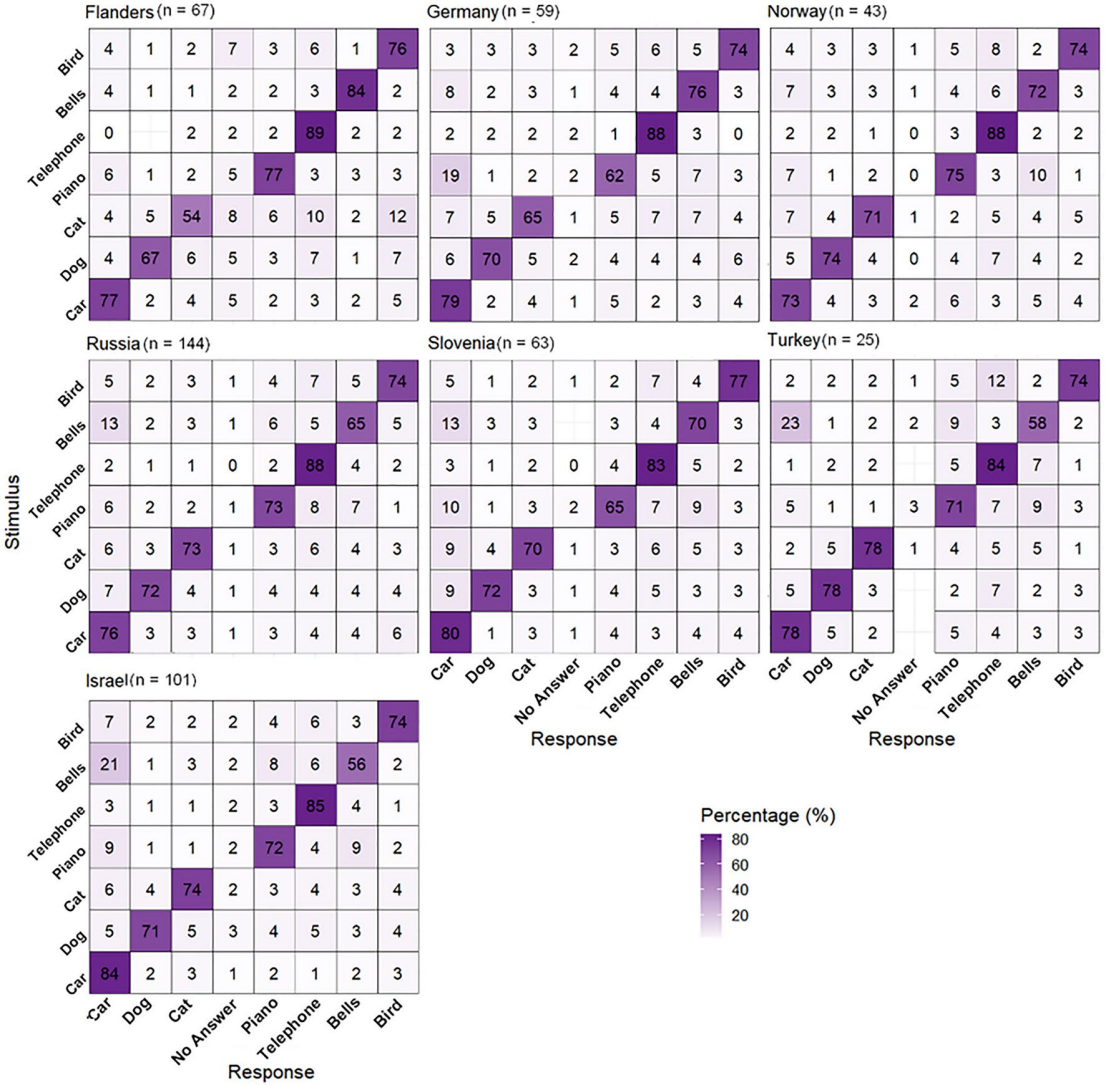
Confusion matrices based on the data of NH children per country in %. The white cells in the confusion matrices show stimulus–response combinations that were never present.

The average SoRT-values per country on the SEC_REF_ are given in Table 2 and shown in Fig. 4. The average values given in Table 2 and shown in Fig. 4 are not taking age into account, which results in slight deviations from the differences between the tests done in different countries estimated with the statistical analyses. Significant differences were determined between SoRTs collected in Flanders and Israel. SoRTs on the SEC_REF_ collected in Israel were significantly poorer than SoRTs collected in Flanders (t (484.8) = 2.3, *p* = 0.021). The difference, corrected for age, was 0.8 dB SNR. None of the other countries showed significant differences with respect to the SoRTs measured (p-values between 0.073 and 0.777). No differences between countries were present in the average SD of the SoRT-values (p-values between 0.096 and 0.666), which was 1.7 ± 0.3 dB SNR in all countries. No difference was determined between children with a different mother tongue compared to children with the country's official language as their mother tongue (t (473.7) = − 1.0, *p* = 0.313). Figure 5 shows the confusion matrices per country. The average across-sound recognition coefficient was 74 ± 5%. The bells were recognized correctly least often (67 ± 10%). The telephone was recognized most often (87 ± 3%). No significant differences were present in the sound-specific recognition scores between countries (p-values between 0.057 and 0.903), except for the bells, which were significantly more difficult in Israel than in other countries (z (8339.4, 18,984) = − 3.4, *p* < 0.001). No significant differences were present between countries in the overall recognition coefficient (χ^2^ (6, N = 18,816) = 3.1, *p* = 0.801).

**Table 2:** SoRT-values per country on the SEC_REF_ of NH children.

Country	Flanders	Germany	Israel	Norway	Russia	Slovenia	Turkey
Average SoRT (dB SNR)	− 8.7 ± 1.9	− 7.7 ± 3.0	− 7.4 ± 2.5	− 8.2 ± 1.6	− 8.0 ± 2.2	− 7.3 ± 2.6	− 8.0 ± 2.0

### Sensitivity and specificity of the optimized SEC-procedures.

**Figure 6:**
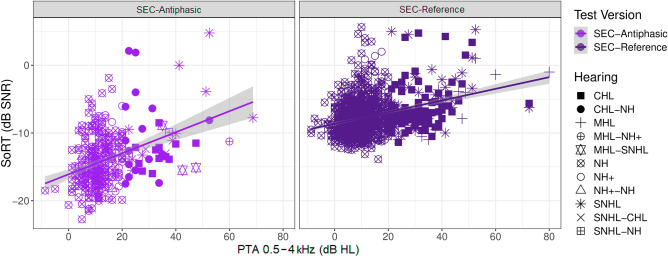
SoRT-values for the SEC_REF_ (Purple) and SEC_APH_ (Pink) in the function of the PTAs_0.5–4 kHz._ The shapes of the points show the type of hearing loss. The hearing groups that only show one type of hearing loss (NH, NH+, CHL, SNHL, and MHL) refer either to the results of the SEC_REF_ done in one ear with that type of hearing or to the SEC_APH_ done by a child with that type of hearing in both ears. No difference was present in how different hearing loss types affect the SoRT-values of the SEC_REF_ or SEC_APH_.

The relation between the SoRT-values on the SEC_APH_ and SEC_REF_ and the PTA_0.5–4 kHz_ is visualized in Fig. 6. PTAs_0.5–4 kHz_ were significantly related to the SoRT-values obtained with the SEC_REF_ (r = 0.07, t (1169) = 9.1, *p* < 0.001) and the SEC_APH_ (r = 0.16, t (3.8, 311) = 7.3, *p* < 0.001). No significant interaction effects were present between the PTA_0.5–4 kHz_ and the type of hearing loss on the SoRT-values obtained with the SEC_REF_ (p-values between 0.210 and 0.820) or the SEC_APH_ (p-values between 0.282 and 0.924)_._

The area under the curve (AUC), optimal pass, fail criteria and sensitivity and specificity are given in Table 3. Both the SEC_REF_ and the SEC_APH_ had very good sensitivity and specificity to detect hearing losses > 40 dB HL. For milder SNHL, the sensitivity and specificity of both tests remained good. SoRT-values obtained with mild CHL showed more variation, visible in Fig. 6, and a slightly lower sensitivity and specificity for mild CHL was obtained (Table 3).

**Table 3:** Values for sensitivity and specificity for CHL, SNHL and combined CHL, SNHL and MHL.

PTA_0.5-4 kHz (dB HL)_	SEC_REF_				SEC_APH_			
	CHL				CHL			
	AUC	Cut-off (dB SNR)	Sens	Spec	AUC	Cut-off (dB SNR)	Sens	Spec
20	0.63 (0.59–0.68)	− 7.9	0.64	0.54	0.69 (0.62–0.76)	− 13.1	0.61	0.67
30	0.69 (0.64–0.76)	− 7.3	0.67	0.68	0.69 (0.59–0.78)	− 13.5	0.69	0.61
40	0.86 (0.79–0.92)	− 6.9	0.92	0.71	0.83 (0.75– 0.93)	− 11.6	1.00	0.77

PTA_0.5-4 kHz (dB HL)_	SNHL				SNHL			
	AUC	Cut-off (dB SNR)	Sens	Spec	AUC	Cut-off (dB SNR)	Sens	Spec
20	0.64 (0.58–0.70)	− 7.8	0.62	0.58	0.74 (0.65–0.81)	− 12.4	0.56	0.72
30	0.82 (0.74–0.90)	− 6.3	0.80	0.80	0.83 (0.72–0.94)	− 11.1	0.80	0.81
40	0.86 (0.75–0.94)	− 6.3	0.93	0.80	0.82 (0.65–0.98)	− 10.1	0.71	0.85

PTA_0.5-4 kHz (dB HL)_	CHL + SNHL + MHL				CHL + SNHL + MHL			
	AUC	Cut-off (dB SNR)	Sens	Spec	AUC	Cut-off (dB SNR)	Sens	Spec
20	0.67 (0.63–0.71)	− 7.8	0.63	0.58	0.72 (0.65–0.78)	− 13.3	0.63	0.67
30	0.74 (0.69–0.79)	− 7.3	0.72	0.68	0.74 (0.66–0.81)	− 13.1	0.69	0.65
40	0.87 (0.81–0.93)	− 6.3	0.89	0.79	0.81 (0.69–0.92)	− 11.6	0.80	0.76
20 (SNHL/MHL) 30 (CHL)	0.70 (0.65–0.75)	− 7.3	0.63	0.68	0.73 (0.65–0.79)	− 13.3	0.68	0.64

The AUC is given with the 90% confidence interval between brackets. The last row gives the sensitivity and specificity of the SEC_REF_ and SEC_APH_ for SNHL/MHL > 20 dB HL & CHL > 30 dB HL, which are the hearing loss degrees for which the cut-off of the SEC_FIX_ was set initially.

**Figure 7:**
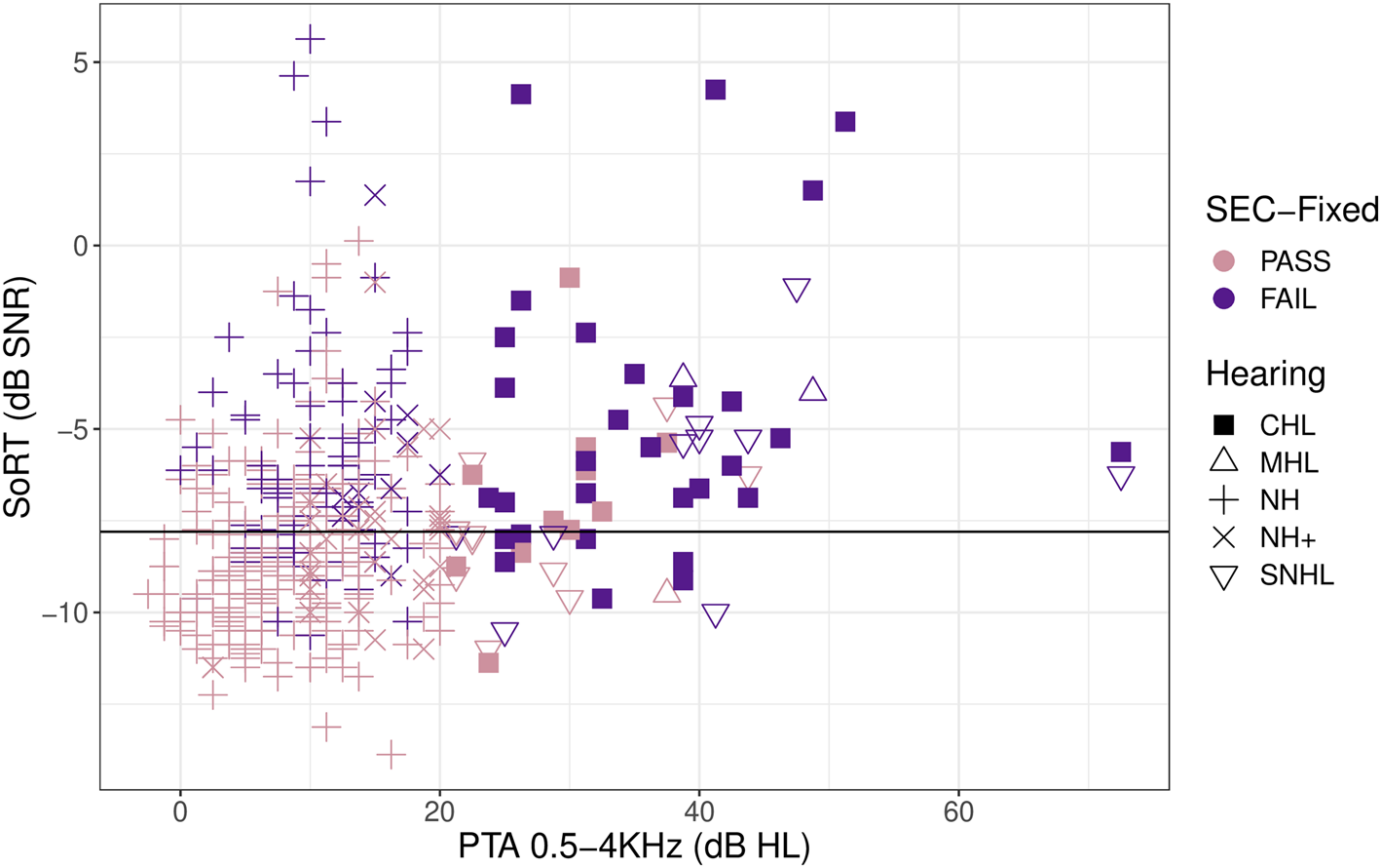
SoRT-values on the SEC_REF_ in function of PTA_0.5–4 kHz_, shapes of points are the hearing type, color visualizes the results on the SEC_FIX_. The SNR used for the SEC_FIX_ is visualized by the horizontal line.

The results of the SEC_FIX_ are visualized together with the SoRT-values of the SEC_REF_ in function of the PTA_0.5–4 kHz_ in Fig. 7. The SEC_FIX_ had a sensitivity and specificity of 71% to pick up the children with either CHL > 30 dB HL and children with MHL/SNHL > 20 dB HL, the hearing loss degrees for which the cut-off was set initially. This was slightly higher than the sensitivity and specificity of the SEC_REF_ for the same hearing loss (Table 3). When comparing the results of the SEC_REF_ and the SEC_FIX_, an SoRT-value of − 7.1 dB SNR on the SEC_REF_ differentiated the best between a pass and a fail result on the SEC_FIX_. Sixty-eight percent of the children scoring poorer SoRT-values than − 7.1 dB SNR on the SEC_REF_ obtained a fail result on the SEC_FIX_, and 76% of the children with SoRT-values lower than − 7.1 dB SNR on the SEC_REF_, obtained a pass result on the SEC_FIX_.

### Minimal hearing loss

**Figure 8:**
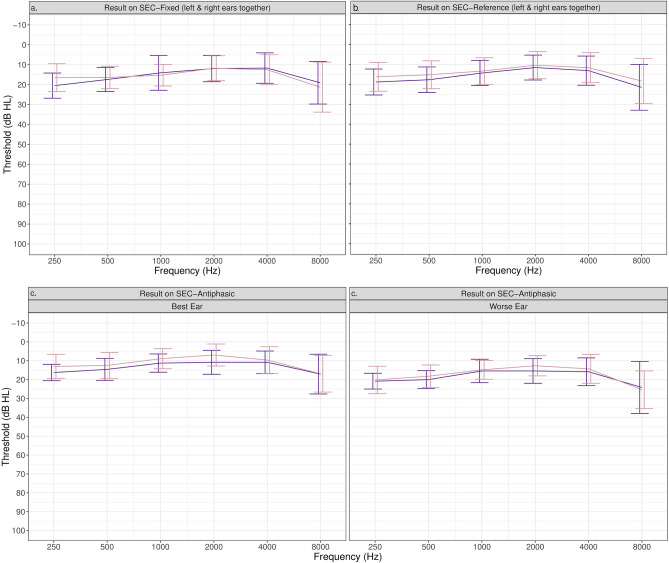
The average thresholds and SD per frequency of children with NH+ with a pass and fail result on the SEC_FIX_ (**a**) and the SEC_REF_ (**b**), and the SEC_APH_ for the best and the worse ear (**c**). For the SEC_REF_ and SEC_FIX_, the results were ear specific, meaning the children could have hearing loss in their other ear. Left and right ears were analyzed together.

Of the 164 ears classified as NH+ tested with the SEC_REF_, 84 (51%) had an SoRT on the SEC_REF_ =  < − 7.8 dB SNR, the optimal cut-off SoRT estimated to detect hearing loss > 20 dB HL. The SoRTs obtained in 80 ears (49%) were > − 7.8 dB SNR. Forty children with NH+ in their worse ear did the SEC_APH_. Twenty-eight children (70%) had an SoRT =  < − 13.3 dB SNR and 12 (30%) had an SoRT > − 13.3 dB SNR on the SEC_APH_, the optimal cut-off SoRT estimated to detect hearing loss > 20 dB HL with the SEC_APH_. Sixty-one ears with NH+ were tested with the SEC_FIX_, in which 21 (34%) a fail-result was obtained and in 40 (66%) a pass result was obtained the SEC_FIX_. The average audiograms of these children, divided into two groups based on their results on the different SEC tests, are given in Fig. 8. For the children who did the SEC_REF_, a significant difference in the thresholds on frequencies 250 Hz (t (142.1) = 2.8, *p* = 0.016) and 500 Hz (t (135.4) = 2.4, *p* = 0.019) was determined between the children with a pass and with a fail result on the SEC_REF_. For the SEC_FIX_, only the threshold at 250 Hz differed significantly (t (44.7) = 2.3, *p* = 0.029). No significant difference in pure tone thresholds was determined between the children with a pass and a fail result on the SEC_APH_ (p-values between 0.075 and 0.960).

## Discussion

The main objective of this study was twofold. The first objective was to investigate whether the test, designed to be culture and language-independent, is truly cultural and language-independent. The second objective was to investigate the sensitivity and specificity of three versions of the SEC, a closed-set tablet-based signal-in-noise self-test, to detect various degrees of CHL, SNHL and MHL in children at school entry.

### Cultural- and language-dependence of the SEC

SoRT-values collected in different countries did agree. The values collected in Israel were 0.8 dB SNR higher than the results collected in Flanders, which was a statistically significant difference. However, compared to the measurement error, which is 1.3 dB SNR^31^, the difference is small and, therefore, clinically not relevant. The confusion matrices, constructed for different countries, showed no differences in overall recognition percentage, indicating that the overall recognizability of the sounds is comparable in all countries. Differences in recognition percentages of the individual sounds were minimal and these differences did not seem to cause clinically relevant changes in SoRT-values or overall recognition percentages. Therefore, adapting the sounds seems unnecessary. No significant differences were determined in the stability of the test, and SoRT-values could be determined with a high stability of 1.3 dB SNR on average in all countries.

### Sensitivity and specificity of the optimized SEC-procedures.

The original SEC with a monaural adaptive procedure before optimization showed high sensitivity and specificity to detect different grades of CHL and SNHL (70–90%)^31^. For hearing loss > 30 dB HL, the sensitivity and specificity of the SEC_REF_ in the current research (70–90%) were comparable with these previous findings. The sensitivity and specificity of the SEC_REF_ for CHL > 30 dB HL (0.67–0.68) were slightly lower than for SNHL (0.80–0.80). However, analyses showed no difference in how different types of hearing loss affect the SoRT-values obtained with the SEC_REF_. Therefore, this difference in sensitivity and specificity was most likely due to differences in the distribution of the PTA_0.5–4 kHz_ -values, in which the average PTA_0.5–4 kHz_ of CHL > 30 dB HL (37.5 ± 7.3 dB HL) was lower than for SNHL/MHL (44.5 ± 10.9 dB HL). For CHL, MHL and SNHL < 30 dB HL, the sensitivity and specificity were slightly lower than what was determined in previous research. Additionally, the pass-fail criteria for the SoRT-values to detect these hearing losses were higher than determined by previous research^31^ indicating that the lower sensitivity and specificity was rather due to NH children scoring worse than expected than due to children with hearing loss scoring better than expected. This may be because of the different circumstances in which the test was conducted. First of all, more children were recruited and tested in hospitals during their already planned visits to the ENT department. It is not unlikely that these children, even though their audiograms were within normal limits, still experienced some hearing difficulties related to the reason for which they initially had their appointments in the ENT department. Moreover, some children were recruited in a private practice for pediatric audiology that sees a lot of children with learning problems in school, which could have complicated the test. As too little quantitative data was collected on the performance of children with developmental disabilities and learning differences to give a substantiated opinion about the usage of the SEC for these children, and this study did not perform any follow-up assessment of the children with pure tone thresholds within normal limits who scored worse than expected on the SEC tests, no conclusion can be made about the reason for their deviating results. Moreover, it is important to note that a normal audiogram does not always mean that functional hearing is normal as well. Therefore, diagnostics and follow-up after a failed SPIN or signal-in-noise test should go beyond performing pure tone audiometry.

For the SEC_APH_, the sensitivity and specificity were similar to slightly lower than the sensitivity and specificity of the SEC_REF_. This contrasts with previous research, where the sensitivity and specificity were higher for antiphasic procedures than for diotic procedures^26^. A possible reason why we did not find higher sensitivity and specificity with the SEC_APH_ than with the SEC_REF_ could be the long training needed for the SEC_APH_ when done by children. Previous research with the SEC indicated that children needed more training when performing an antiphasic test than a monaural test^31^. Therefore, in this study, an extra training phase with 21 trials was provided before the test phase. However, our results indicate that children who performed the SEC_REF_ before the SEC_APH_ still scored better on the SEC_APH_, showing that the extra training phase was still insufficient. As the results obtained with the SEC_APH_ as the first in the sequence were 2 dB SNR worse than those obtained with the SEC_APH_ done second in the sequence, extra variance was induced, possibly reducing the sensitivity and specificity. However, even if higher sensitivity and specificity would be obtained when only considering SoRT-values of SEC_APH_ performed as the second test in the sequence, this is somewhat irrelevant as it would practically be almost impossible to perform an even longer training phase for the SEC_APH_ when using it as a systematic screening test. Previous research with antiphasic procedures done with adults does not report the length of the training done^26,27^. Therefore, it is unclear whether longer training is only needed when testing children. Another possible reason for the high number of children with normal audiograms but with high SoRTs on the SEC_APH_ is the development of binaural hearing during childhood^35^. Research shows that the binaural processing of temporal fine structure is not fully developed in children aged 5 years, 6 months to 9 years, 4 months, which can limit the binaural unmasking needed to pass the SEC_APH_^35^. Developmental effects on the SoRT-values of the SEC_APH_ can cause additional variance in these results, which can lower the sensitivity and specificity of the test for children, explaining why an antiphasic procedure is less suitable for children than for adults.

The sensitivity and specificity of the SEC_FIX_ to detect CHL > 30 dB HL and MHL and SNHL > 20 dB HL were slightly higher than the sensitivity of the SEC_REF_ and the SEC_APH_ for the same type of hearing loss. However, when comparing the results on the SEC_REF_ and the SEC_FIX_, it is important to notice that, even though the SEC_FIX_ presented all the sounds at an SNR of − 7.8 dB SNR, the best cut-off SoRT to differentiate with the SEC_REF_ between children obtaining a pass and a fail result on the SEC_FIX_, was − 7.1 dB SNR. This indicates that the SEC_FIX_ was slightly more difficult than the SEC_REF_. A possible reason is that when a child has an SoRT close to the SNR used for the SEC_FIX_, all the sounds played were challenging and required full attention. Moreover, the SEC_FIX_ allows for very few accidental mistakes, as when one sound is answered wrongly, the chance to get a pass result already drops by at least 5% (1/21). Therefore, the SEC_FIX_ can be more sensitive to attention dwells, which can result in more fail results, especially for the children with SoRTs close to the SNR used in the SEC_FIX_.

Within this research, supplementary analyses were performed on children with PTA_0.5–4 kHz_ < 20 dB HL but with one or more elevated thresholds. In our research, around 30–50% of these children failed on the different versions of the SEC when using the optimal cut-offs to differentiate NH children from children with mild CHL, SNHL or MHL ≥ 20 dB HL, which indicates functional hearing problems. For the SEC_REF_, this percentage was higher than the percentage of fail results in the NH group when using the same cutoff-value (NH+: 49%, NH: 42%), for the SEC_APH_ and the SEC_FIX_, this percentage was in line with the percentage of fail results obtained for the NH group when using the same cutoff-value (± 30%). The frequencies at which the thresholds were elevated did not seem to be related to whether or not they failed the SEC tests. The relation determined between minimal hearing loss and functional hearing problems aligns with previous research showing that even minimal hearing loss can affect functional hearing abilities^6^, which, again, favors qualitative follow-up after failing a functional hearing test, even though the problems determined with only pure tone audiometry are minimal.

## Conclusion

High sensitivity and specificity of the SEC_REF,_ SEC_FIX_ and SEC_APH_ for mild hearing loss were determined. 30–50% of the children with one or more elevated thresholds, but a PTA_0.5–4 kHz_ within normal limits were detected with the SEC tests as well. For the SEC_APH,_ children seemed to need more than twice the length of the training needed for monaural tests before stable SoRT-values could be obtained. For that reason, this research favors the use of monaural test procedures for children, such as the SEC_REF_ and the SEC_FIX_. Previous research showed a significantly shorter test duration for the SEC_FIX_ than for the SEC_REF_^31^. Therefore, the SEC_FIX_ can be particularly useful for screening children when test efficiency is of major importance, while the SEC_REF_ can be useful when an SoRT-value needs to be obtained. Differences determined between countries were negligible, indicating cultural and language independence. Therefore, the SEC has great potential to be used as an international hearing screening test for young children at school entry, regardless of the resources available to develop and implement hearing screening programs. Consequently, the SEC can assist in the early detection of late-onset, acquired or progressive hearing loss in children internationally.

## Data Availability

The data that support the findings of this study are available from the corresponding author, E.V.d.B., upon reasonable request.

## References

[1] Butcher E, Dezateux C, Cortina-Borja M, Knowles RL (2019). Prevalence of permanent childhood hearing loss detected at the universal newborn hearing screen: Systematic review and metaanalysis. PLoS ONE.

[2] Fortnum H, Summerfield AQ, Marshall DH, Davis AC, Bamford JM (2001). Prevalence of permanent childhood hearing impairment in the United Kingdom and implications for universal neonatal hearing screening: questionnaire based ascertainment study. BMJ.

[3] Bess FH, Dodd-Murphy J, Parker RA (1998). Children with Minimal sensorineural hearing loss; Prevalence, educational performance, and functional status. Ear Hear..

[4] Porter H, Sladen DP, Ampah SB, Rothpletz A, Bess FH (2013). Developmental outcomes in early school-age children with minimal hearing loss. Am. J. Audiol..

[5] Winiger A, Alexander J, Diefendorf A (2016). Minimal hearing loss: from a failure-based approach to evidence-based practice. Am. J. Audiol..

[6] Moore DR, Zobay O, Ferguson MA (2020). Minimal and mild hearing loss in children. Ear Hear..

[7] Briscoe J, Bishop DVM, Norbury CF (2001). Phonological processing, language, and literacy: A comparison of children with mild-to-moderate sensorineural hearing loss and those with specific language impairment. J. Child Psychol..

[8] Lewis DE, Valente DL, Spalding JL (2015). Effect of minimal/mild hearing loss on children’s speech understanding in a simulated classroom. Ear Hear..

[9] Yoshinaga-itano C, Coulter D, Thomson V (2001). Developmental outcomes of children with hearing loss born in Colorado hospitals with and without universal newborn hearing screening programs. Semin. Neonatal..

[10] McCann DC (2009). Reading and communication skills after universal newborn screening for permanent childhood hearing impairment. Arch. Dis. Child..

[11] Kennedy C (2007). Language ability after early detection of permanent childhood hearing impairment. Community Ear Hear. Health.

[12] Pimperton H, Kennedy CR (2012). The impact of early identification of permanent childhood hearing impairment on speech and language outcomes. Arch. Dis. Child..

[13] Cupples L (2018). Spoken language and everyday functioning in 5-year-old children using hearing aids or cochlear implants. Int. J. Audiol..

[14] Yong M (2019). Cost-effectiveness of school hearing screening programs: A scoping review. Am. J. Otolaryngol. Head Neck Med. Surg..

[15] World Health Organization. Hearing screening: considerations for implementation. In *Licence: CC BY-NC-SA 3.0 IGO* (2021).

[16] Sharma R (2022). An economic evaluation of Australia’s Newborn hearing screening program: A within-study cost-effectiveness analysis. Ear Hear..

[17] Neumann K, Mathmann P, Chadha S, Euler HA, White KR (2022). Newborn hearing screening benefits children, but global disparities persist. J. Clin. Med..

[18] Wouters, J. *et al.* EFAS Working Group on school age hearing screening. In *EFAS Working Group on school age hearing screening* (2017).

[19] Mehra S, Eavey RD (2009). The epidemiology of hearing impairment in the United States: Newborns, children, and adolescents. Otolaryngol. Neck Surg..

[20] Fletcher H, Galt RH (1950). The perception of speech and its relation to telephony. J. Acoust. Soc. Am..

[21] French NR, Steinberg JC (1947). Factors governing the intelligibility of speech sounds. J. Acoust. Soc. Am..

[22] Denys S (2019). Language-independent hearing screening based on masked recognition of ecological sounds. Trends Hear..

[23] Smits C, Kapteyn TS, Houtgast T (2004). Development and validation of an automatic speech-in-noise screening test by telephone. Int. J. Audiol..

[24] Smits C, Goverts S, Festen JM (2013). The digits-in-noise test: Assessing auditory speech recognition abilities in noise. J. Acoust. Soc. Am..

[25] Smits C (2017). Improving the efficiency of speech-in-noise hearing screening tests. Ear Hear..

[26] de Sousa KC, Swanepoel DW, Moore DR, Myburgh HC (2020). Improving sensitivity of the digits-in-noise test using antiphasic stimuli. Ear Hear..

[27] Smits C, Watson CS, Kidd GR, Moore DR, Goverts ST (2016). A comparison between the Dutch and American- English digits-in-noise (DIN) tests in normal-hearing listeners. Int. J. Audiol..

[28] Plomp R (1978). Auditory handicap of hearing impairment and the limited benefit of hearing aids. J. Acoust. Soc. Am..

[29] Levitt H (1971). Transformed up-down methods in psychoacoustics. J. Acoust. Soc. Am..

[30] George ELJ, Festen JM, Goverts TS (2012). Effects of reverberation and masker fluctuations on binaural unmasking of speech. J. Acoust. Soc. Am..

[31] Van den Borre E (2022). Language-independent hearing screening—Increasing the feasibility of a hearing screening self-test at school-entry. Trends Hear..

[32] Perkins E (2021). Further evidence for the expansion of adult cochlear implant candidacy criteria. Otol. Neurotol..

[33] RStudio. RStudio: Integrated Development for R. (2020).

[34] Metz CE (1978). Basic principles of ROC analysis. Semin. Nucl. Med..

[35] Flanagan SA (2021). Development of binaural temporal fine structure sensitivity in children. J. Acoust. Soc. Am..

